# Risk factors for secondary transmission of *Shigella* infection within households: implications for current prevention policy

**DOI:** 10.1186/1471-2334-12-347

**Published:** 2012-12-12

**Authors:** Lian Boveé, Jane Whelan, Gerard JB Sonder, Alje P van Dam, Anneke van den Hoek

**Affiliations:** 1 Department of Infectious Diseases, Public Health Service (GGD) Amsterdam, Nieuwe Achtergracht 100, PO Box 2200, Amsterdam, 1000 CE, the Netherlands; 2 Department of Internal Medicine, Academic Medical Center, Division of Infectious Diseases, Tropical Medicine and AIDS, Meibergdreef 9, Amsterdam, 1105 AZ, The Netherlands; 3 Public Health Laboratory, Public Health Service (GGD) Amsterdam, Nieuwe Achtergracht 100, PO Box 2200, Amsterdam, 1000 CE, the Netherlands; 4 Department of Medical Microbiology, Onze Lieve Vrouwe Gasthuis (OLVG Hospital), Postbus 95500, Amsterdam, 1090 HM, the Netherlands

**Keywords:** Shigella, Infection control, Community-acquired infections/epidemiology, Disease transmission, Infectious, Child, Preschool

## Abstract

**Background:**

Internationally, guidelines to prevent secondary transmission of *Shigella* infection vary widely. Cases, their contacts with diarrhoea, and those in certain occupational groups are frequently excluded from work, school, or daycare. In the Netherlands, all contacts attending pre-school (age 0–3) and junior classes in primary school (age 4–5), irrespective of symptoms, are also excluded pending microbiological clearance. We identified risk factors for secondary *Shigella* infection (SSI) within households and evaluated infection control policy in this regard.

**Methods:**

This retrospective cohort study of households where a laboratory confirmed *Shigella* case was reported in Amsterdam (2002–2009) included all households at high risk for SSI (i.e. any household member under 16 years). Cases were classified as primary, co-primary or SSIs. Using univariable and multivariable binomial regression with clustered robust standard errors to account for household clustering, we examined case and contact factors (*Shigella* serotype, ethnicity, age, sex, household size, symptoms) associated with SSI in contacts within households.

**Results:**

SSI occurred in 25/ 337 contacts (7.4%): 20% were asymptomatic, 68% were female, and median age was 14 years (IQR: 4–38). In a multivariable model adjusted for case and household factors, only diarrhoea in contacts was associated with SSI (IRR 8.0, 95% CI:2.7-23.8). In a second model, factors predictive of SSI in contacts were the age of case (0–3 years (IRR_case≥6 years_:2.5, 95% CI:1.1-5.5) and 4–5 years (IRR_case≥6 years_:2.2, 95% CI:1.1-4.3)) and household size (>6 persons (IRR_2-4 persons_ 3.4, 95% CI:1.2-9.5)).

**Conclusions:**

To identify symptomatic and asymptomatic SSI, faecal screening should be targeted at all household contacts of preschool cases (0–3 years) and cases attending junior class in primary school (4–5 years) and any household contact with diarrhoea. If screening was limited to these groups, only one asymptomatic adult carrier would have been missed, and potential exclusion of 70 asymptomatic contacts <6 years old from school or daycare, who were contacts of cases of all ages, could have been avoided.

## Background

Shigellosis (bacillary dysentery) is an acute intestinal infection caused by the toxin-producing gram-negative bacterium *Shigella*. The route of infection is faecal-oral, via the hands or through ingestion of contaminated food or water. The incubation period is typically 1–3 days. Clinical symptoms include fever, watery diarrhoea, abdominal cramps and bloody, slimy stools [1]. Disease is most severe and the case-fatality rate highest in children, the elderly and those who are immunocompromised. Case-fatality rate depends on the serotype, and is up to 20% of patients hospitalised with *S. dysenteriae* which occurs predominantly in less industrialised countries. In industrialised countries, *S. sonnei* and *S. flexneri* account for the majority of cases, and in the Netherlands about 75% of infections are imported, most frequently in the summer months [2]. Nationally, 300–600 cases of bacillary dysentery are reported each year, yielding an approximate annual incidence of 3.2/100,000 population [2]. Secondary attack rates in households can be high [3] and infections are associated with significant morbidity and socioeconomic cost as infected individuals may be excluded from school or work pending microbiological clearance.

Guidelines for contact tracing and the control of shigellosis differ across jurisdictions. In Australia, contacts are screened routinely only in outbreak situations [4]. They are excluded from attending work or childcare, whether symptomatic or not, if they are in risk groups such as food handlers, carers or children attending childcare, until 2 successive stool samples collected a minimum of 24 hours apart are negative. In the USA, it is recommended that only symptomatic attendees and staff members in childcare centres where *Shigella* infection has been identified should have a faecal specimen cultured [5]. Children and staff can generally return to the child care facility ≥24 hours after they are symptom free. In some US states, exclusion is continued until results of 2 stool cultures are negative for *Shigella* species. In the UK, contacts in risk groups are screened routinely, but microbiological clearance (two negative faecal specimens taken at intervals ≥48 hours) is required for cases of S. *dysenteriae*, S. *flexneri* or S. *boydii*, but not for cases of S. *sonnei*[6].

In the Netherlands, shigellosis is notifiable by law [7]. When a case is identified, it is the responsibility of the Public Health Service (PHS) to trace contacts in order to prevent secondary infection. Until 2001, it was national policy to screen all household contacts of a *Shigella* case. This policy was amended based on the results of a retrospective study of shigellosis cases and their contacts reported from 1991–1998 in Amsterdam, which concluded that the highest risk of secondary transmission and hospitalisation was among children under 16 years [8]. It was recommended that contact tracing should specifically be targeted at households where children reside (hereafter, “high risk households”). In 2001, national guidelines were adjusted accordingly [9]. Since then, contact tracing is limited to faecal sampling of all household contacts if the primary case is younger than 16 years or one or more contacts in the family are younger than 16 years. If the primary case is older than 16 years and there are no younger contacts in the family, selective faecal sampling of family contacts that are care-workers or food-handlers, and those that have symptoms consistent with a *Shigella* infection is conducted. Under current guidelines, cases who attend childcare centres (0–3 years), or those in junior classes in primary school (4–6 years) are excluded until two consecutive faecal samples, taken at least 3 days apart and 48 hours after completion of antibiotic therapy, are confirmed negative. Furthermore, it is recommended that children of the same age who are contacts of a shigesllosis case of any age, should also be excluded from school (irrespective of symptoms) until one faecal sample is confirmed negative for *Shigella*[9]. The aim of this study was to determine the proportion of secondary transmissions in “high risk” households and the characteristics of primary cases and their contacts that are associated with secondary transmission, thereby to evaluate the appropriateness of current exclusion policies in relation to young children.

## Methods

### Routine surveillance data

A confirmed case of *Shigella* infection was defined as any person from whom a *Shigella* species was isolated from a faecal sample, reported to the PHS of Amsterdam from 2002 to 2009. In addition, case data routinely collected included age, gender, occupation, country of birth, dates of departure and return on any recent foreign trips, date of onset of illness and information about hospitalisation.

### Household contact study

This was a retrospective cohort study including all occupants of “high risk” households in which a primary case of *Shigella* infection was reported to the PHS of Amsterdam from 2002 to 2009. A primary case was the first person in a high risk household to present with laboratory confirmed *Shigella* infection in a faecal sample. A high risk household was any household with more than one inhabitant including at least one child <16 years, where a primary case (of any age) stayed for at least one overnight, using shared toilet facilities, from the onset of symptoms to the date of notification to the PHS. Outcomes of interest were (a) any laboratory confirmed secondary infection and (b) asymptomatic laboratory confirmed secondary infection. Contacts were asked to report any symptoms experienced (diarrhoea, fever), and on what day they began relative to the primary case. For comparative purposes and to be consistent with previous research [8], secondary infection was defined as laboratory confirmed *Shigella* infection in a household contact developed >1 day after the primary case. If a symptomatic contact’s first day of illness was ≤1 day after the primary case then this contact was considered a co-primary infection and was excluded from the study. Primary cases and their contacts were also excluded if their most likely source was “Men who have sex with men” (MSM) or if the source was a common exposure to the same suspected food-source. In accordance with current guidelines, contacts were also asked to provide a faecal sample for culture by the PHS Regional Laboratory of Amsterdam, in addition to provision of general demographic information. As this research was conducted in the context of routine surveillance, no ethical approval was required.

### Laboratory methods

For diagnosis of shigellosis, faecal specimens were suspended in saline and plated onto Hecto-en enteric agar. Green colonies, suspected for *Shigella*, were tested for fermentation of glucose and lactose using a TSI-slant and tested for urease production. Urease-negative, glucose-fermenting, lactose-nonfermenting strains were subsequently determined to the species level using API-20E tests (Biomerieux, Craponne, France) and agglutinated with polyvalent antisera against *S.sonnei, S.flexneri, S.boydii* and *S.dysenteriae*.

### Statistical analysis

Data were analysed using Stata 11 (StataCorp LP, College Station, TX, USA). The proportion of secondary infections was the number of laboratory confirmed infected contacts divided by the total number of household contacts tested. We hypothesized that both individual characteristics of the contact (age, sex, whether symptomatic or not, hospitalized or not) as well as contextual factors in the household (household size, characteristics of the case in the household - age and sex, *Shigella* serotype, ethnicity, whether hospitalized or not) could be associated with secondary transmission. Age was classified in three age-groups based on school attendance: those aged 0–3 years attending pre-school, those in junior classes in primary school aged 4–5 years, and those aged ≥6 years attending senior classes in primary school. Duration from date of onset of illness to date of notification was used as a proxy measure of duration of transmission risk (i.e. prior to receipt of hygiene advice from a health professional). Univariable associations between the outcome, and individual and contextual characteristics within the household, were first tested using the Chi-squared test or Fischer’s exact test. As contacts and cases were clustered within households and there were a large number of clusters relative to the total sample of contacts, we used ordinary univariable and multivariable binomial regression models to obtain risk ratios with 95% confidence intervals. These were corrected for correlation between individuals within households using clustered robust standard errors based on the Huber-White sandwich estimator [10,11]. If the univariable association was significant at p<0.1, variables were included in the multivariable analysis. Missing values were excluded. Finally, to compare with previous research, the annual incidence of shigellosis was estimated as the number of positive shigellosis cases per year per 100,000 residents of Amsterdam.

## Results

### Routine surveillance data

From 2002 to 2009, 420 primary cases of shigellosis were reported through routine surveillance. The median age was 33 years (interquartile range: 26–42) and 57% (n=241) were male. Eighteen percent of cases (n=76) were MSM and 5 cases acquired their infection from a common suspected food-source. Of non-MSM cases born in the Netherlands, 82% (n=155/329) acquired their infection abroad. Of non-Dutch cases who had recently travelled abroad (n=90), 50% (n=45) contracted the infection while visiting their country of origin. The most common countries where infection was acquired were Morocco (n = 52), Egypt (n = 38), India (n=31), Ghana (n = 14), Indonesia (n = 10). Overall, 31% (n=130) of infections were reported in August and September. All primary cases had diarrhoea and 15% (n=63) were admitted to hospital. There were no deaths among cases.

### Household study: characteristics of cases, contacts and households

**Figure 1:**
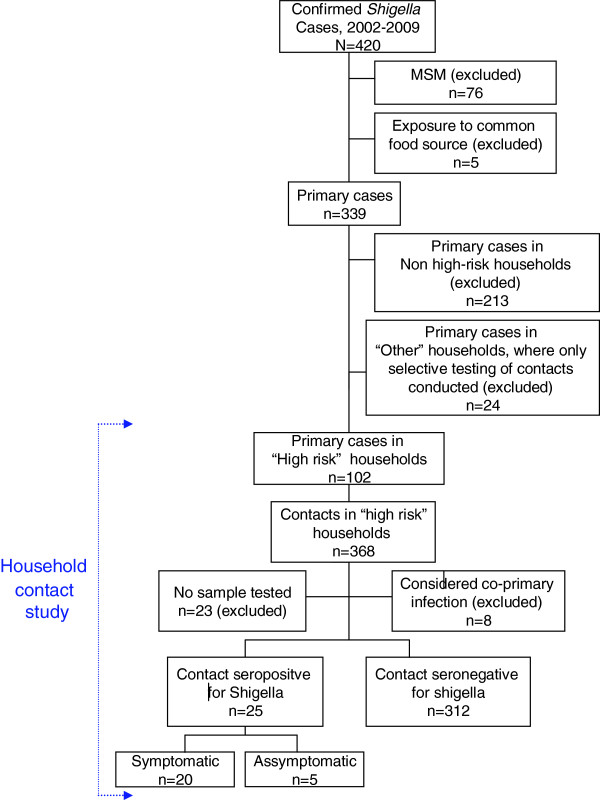
Flow chart of study population.

MSM and those who were exposed to a common food source were excluded from the household study. Of the remaining 339 primary cases, 213 resided in non high-risk households and 24 had contacts that were selectively screened because they were symptomatic or were working as food-handlers or care-givers (Figure 1). Notably, no secondary transmissions occurred in these households. Ultimately 368 contacts related to 102 primary cases in 102 high risk households were identified. All were offered screening and 23 did not participate (Figure 1) and 8 were considered co-primary infections and were also excluded from the household study. The median household size was 4 persons (Interquartile Range,IQR: 3–5): 61 households contained 2–4 people, 24 households 5–6 people, and 17 households, >6 people. A greater proportion of cases (35%) compared to contacts (23%) were under 6 years old cases (Pearson chi-squared, p=0.03). The gender distribution was similar. Baseline characteristics of cases and household contacts are presented in Table 1.

**Table 1:** **Baseline characteristics of primary *Shigella *cases (n=102) and related contacts (n=337) in high risk households, Amsterdam, The Netherlands 2002–2009**

**Characteristics**		**Primary cases (n=102)**		**Household contacts (n=337)**		**p value**
		**n**	**%**	**n**	**%**	
Age group						
	0–3	20	20	48	14	
	4–5	15	15	19	9	
	>6	67	66	260	77	0.030
Gender						
	female	53	52	171	51	
	Male	49	18	166	49	0.802
Country of birth						
	Netherlands	30	29	91	27	
	Western, other	3	3	6	2	
	Non-western	70	68	236	71	
Diarrhoea						
	Yes	102	100	87	26	
	No	0	0	228	68	
	Unknown	0	0	22	7	
Shigella isolated						
	Yes	102	100	25	7	
	No	0	0	312	86	
Shigella serotype						
	S. sonnei	57	56	15	4	
	S. flexneri	36	35	8	2	
	S. boydii	6	6	2	1	
	S. dysenteriae	3	3	0	0	
	Negative	0	0	312	93	
Months reported						
	Dec-Jan	9	9	46	14	
	Feb-Mar	6	6	15	4	
	Apr-May	9	9	25	7	
	Jun-Jul	8	8	20	6	
	Aug-Sep	51	50	168	50	
	Oct-Nov	19	19	63	19	
Hospitalised						
	No	22	22	1	0	
	Yes	80	78	336	100	

### Household study: risk factors for secondary transmission

*Shigella* was isolated from 25 of the remaining 337 contacts (7.4%) of whom 68% (n=17) were female, and the median age was 14 years (IQR: 4–38). The attack rate ranged from 6% in contacts aged 0–3 years, to 17% in 4–5 year old contacts (Table 2). Ethnic backgrounds of those who acquired secondary infection were Moroccan (n=12, 48%), Dutch (n=5, 20%), Surinamese (n=2, 8%) Turkish (n=2, 8%) Croatian (n=2, 8%) or “other” (n=2, 8%). One 8-month old contact was admitted to hospital and there were no deaths. Over half of secondary infections (56%, n=14) were associated with primary cases who were under 6 years old. The nature of the positive contact’s relationship to the primary case was as follows: sibling (n=7, median age 4, IQR: 2–12), mother (n=7), father (n=3), offspring (n=2), or other family contact (n=6, median age 5.5, IQR: 4–14). Statistically there was no association between secondary infection and the nature of the contact’s relationship to the primary case, and we did not find any difference in age between siblings who were secondary versus non-secondary cases (mean age 8.1 & 10.6 respectively, p=0.502). Thirteen primary cases were associated with one positive household contact and 6 primary cases were associated with 2 positive household contacts. No factor was identified that was associated with >1 secondary transmission. Overall, the mean annual incidence of *Shigella* infection in Amsterdam from 2002–2009 was 7.7/100,000 persons.

**Table 2:** Univariable and multivariable risk factors for secondary *Shigella *transmission to 337 contacts within 102 households*, Amsterdam, The Netherlands 2002–2009

**Exposure**		**Total no. of household contacts**	**Any secondary transmission**			**Univariable**				**Multivariable**		
		**N**	**n**	**%**	**RR**	**95% CI**		**p value**	**RR**	**95% CI**		**p value**
						**Lower**	**Upper**			**Lower**	**Upper**	
***Contact characteristics (n=337 contacts)***												
Total		337	25	7.4	n/a	n/a	n/a	n/a	n/a	n/a	n/a	n/a
Age-group												
	≥ 6 years	260	17	6.5	ref.	ref.	ref.	ref.	ref.	ref.	ref.	ref.
	0–3	48	3	6.3	1.0	0.3	3.3	0.943	1.0	0.3	3.2	0.979
	4–5	29	5	17.2	2.6	1.0	6.8	0.046	1.3	0.6	3.0	0.527
Gender												
	Female	171	17	9.9	ref.	ref.	ref.	ref.				
	Male	166	8	4.8	0.5	0.2	1.2	0.109				
Country of birth												
	Netherlands / Other Western	97	7	7.2	ref.	ref.	ref.	ref.				
	Other	236	18	7.6	1.1	0.5	2.3	0.890				
Diarrhoea												
	No	228	5	2.2	ref.	ref.	ref.	ref.	ref.	ref.	ref.	ref.
	Yes	87	20	23.0	10.5	3.7	29.9	<0.001	8.0	2.7	23.8	<0.001
Hospitalised												
	No	336	24	7.1	ref.	ref.	ref.	ref.				
	Yes	1	1	100.0	-	-	-	-				
***Household factors (n=102 households)***												
Household size												
	2–4 persons (in 61 households)	128	5	3.9	ref.	ref.	ref.	ref.	ref.	ref.	ref.	ref.
	5–6 persons (in 24 households)	96	5	5.2	1.3	0.4	4.8	0.658	1.4	0.4	4.6	0.570
	>6 persons (in 17 households)	113	15	13.3	3.4	1.3	8.7	0.011	3.1	0.9	10.1	0.064
Age of primary case in household												
	≥ 6 years	218	11	5.1	ref.	ref.	ref.	ref.	ref.	ref.	ref.	ref.
	0–3	58	6	10.3	2.1	0.8	5.5	0.152	2.6	1.0	7.1	0.061
	4–5	61	8	13.1	2.6	1.2	5.7	0.018	1.9	0.8	4.5	0.163
Gender of primary case in household												
	Female	174	9	5.2	ref.	ref.	ref.	ref.				
	Male	163	16	9.8	1.9	0.9	4.1	0.103				
Primary case in household hospitalised												
	No	264	21	8.0	ref.	ref.	ref.	ref.				
	Yes	73	4	5.5	1.0	0.9	1.2	0.405				
Time from date of onset to notification of primary case												
	≤1 week	95	9	9.5	ref.	ref.	ref.	ref.				
	1–3 weeks	146	8	5.5	0.6	0.2	1.5	0.262				
	>3 weeks	96	8	8.3	0.9	0.4	2.0	0.761				

*The unit of analysis is the individual contact (n=337) and cluster characteristics are at the household level: household size, age and gender of the primary case in the household, whether they were hospitalised and time from date of onset to notification of primary case, as above.

At the univariable level (Table 2), secondary transmission was more likely to occur if the household contact was aged 4–5 years or if they had diarrhoea, or in turn if the primary case was aged 4–5 years old, or in large households (>6 persons). In the multivariable model, the only significant correlation observed, was between secondary infection and the presence of diarrhoea in the household contact. We re-ran the multivariable model without diarrhoea as a variable (data not shown in Table 2). Independent predictors of secondary infection in contacts were then if the case was aged 0–3 years (Incidence Rate Ratio, IRR_case≥6 years_:2.5, 95% CI:1.1-5.5) or 4–5 years (IRR_case≥6 years_:2.2, 95% CI:1.1-4.3) and households with more than 6 persons (IRR2-4 persons 3.4, 95% CI:1.2-9.5). Contact characteristics were not significant in this model. Overall, 5/25 secondary infections were asymptomatic (20%). These were persons aged 5 and 8 years old, and three were aged >30 years. When contacts who were symptomatic and positive for Shigella were excluded (n=20), we did not find any association between the age of the contact (≥6years old (ref.) versus <6 years) and the risk of asymptomatic infection (IRR:0.9, 95% CI:0.2-5.0).

## Discussion

The proportion of secondary transmissions of laboratory confirmed *Shigella* infection in high risk household contacts of primary cases reported in Amsterdam from 2002 to 2009 was 7.4%. Though not directly comparable to our study, similar intra-familial or household secondary transmission rates of *Shigella* have been reported in studies conducted in outbreak settings internationally [12,13]. This rate is also similar to that of 8% reported by Vermaak et al. [8] in Amsterdam from 1992–1998. In our study, only households with contacts considered to be at high risk of secondary infection were included. As this represented a smaller denominator population than in Vermaak et al. [8], we had expected to find a relative increase in the rate of secondary infection. One explanation is that we underestimated the secondary attack rate and that (unscreened) positive asymptomatic cases in non-high risk households were missed. We consider this unlikely as in Vermaak et al. [8] this accounted for only 3 extra cases over 8 years. An alternative explanation is that hygiene standards in households in the Netherlands and abroad have improved over time, reducing the potential for secondary spread. The majority of *Shigella* infections are imported, but recent national research has shown that between 1995 and 2006 there was a significant reduction in the incidence of *Shigella* infection among travelers from the Netherlands which was related to improved hygiene standards in the countries visited [14]. Despite a doubling in the annual number of travelers to (sub)tropical countries from about 1 million in 1999 to 2 million in 2007 [15], the incidence of shigellosis in Amsterdam has remained relatively static at 8/100,000 in 1998 [8], and 7.7/100,000 annually from 2002–2009.

Where outbreaks of shigellosis have occurred in nurseries and schools, they have generally been attributable to children with diarrhoea who visited the institution or those who returned to school before being culture-confirmed negative [12,16]. Outbreaks have been brought under control by excluding young shigellosis cases from school or daycare where supervision of a child’s hygiene may be inconsistent, pending microbiological clearance [17,18]. In our first multivariable model, the only factor independently associated with *Shigella* positivity in a household contact was diarrhoea, irrespective of the age of the case or of the contact. The current policy, that all contacts with diarrhoea should be investigated for the presence of the bacterium *Shigella*, is therefore supported.

In the second multivariable model which examined predictors of secondary infection in contacts, preschool cases aged 0–3, those in junior classes in primary school aged 4–5 years, and those in large households were more likely to transmit (both symptomatic and asymptomatic) infection. Typically, young children who use the toilet independently but have limited understanding of good hand- and toilet-hygiene may be particularly susceptible to transmitting secondary infection. We did not find any increased risk among siblings of cases, or their mothers compared to other household contacts however, unlike similar research examining household transmission of E.Coli 0157 [19]. Based on our findings, screening of all contacts of cases who are under 6 years is also recommended. In fact, had faecal screening been limited to household contacts of cases who were under 6 years old and contacts with diarrhoea as we suggest, 96% of secondary cases would have been detected and only one asymptomatic adult carrier would have been missed. Additional faecal sampling of 164 contacts would not have been required.

The policy in the Netherlands of excluding all contacts under 6 years old pending a single negative faecal culture sample is generally not supported by our findings. In the multivariable models, the age of the contact was not independently associated with secondary *Shigella* infection and we found no association between young age of contact (<6 years old) and a risk of asymptomatic infection. In our study over the 8 year period, 70 asymptomatic children under 6 years old were potentially excluded from school or daycare pending microbiological clearance. This yielded only one asymptomatic infection. Although a formal cost-benefit analysis would be necessary to systematically compare costs, given considerable practical difficulties and low added value, the policy of excluding young children who are asymptomatic contacts of a case with shigellosis should be revisited.

There were a number of study limitations: firstly, we were unable to examine the risk of asymptomatic secondary transmission in low risk households, however among those at highest risk in these households who were screened (i.e. those who were symptomatic, or were care-workers or food-handlers) no secondary transmissions occurred. Secondly, we defined a secondary infection in a household contact as one that developed >1 day after the primary case. Had we used a more conservative definition (e.g. ≥3 days, based on the median incubation period), one additional case would have been reclassified, representing a secondary attack proportion of 7.2%. The associations at both univariable and multivariable level would not change however. Thirdly, there was a delay between date of onset of illness and date of notification of >3 weeks in 28% of cases. Recall bias is therefore likely, and cases and contacts may have reported estimated rather than precise dates of onset of illness. Ultimately, delay in reporting was not associated with secondary transmission of infection. Fourthly, culture was used for diagnosis of shigellosis, though it is recommended that the sample is submitted within 24 hours, false negatives may have occurred. The use of more sensitive molecular methods [20] might have revealed more cases of secondary transmission. Finally, given the low proportion of secondary transmissions, it is possible some differences may have been undetected due to insufficient power.

## Conclusions

In conclusion, guidelines relating to screening and exclusion of contacts in order to prevent secondary transmission of *Shigella* infection vary widely internationally. Prevention of secondary transmission through education and promotion of hand washing and strict hygiene practices in affected households remain the mainstay of *Shigella* control [5,6,21]. To identify symptomatic and asymptomatic SSI, all household contacts of young *Shigella* cases (<6 years old) and contacts with diarrhoea should be screened. Exclusion of cases and contacts with diarrhoea from work, school and daycare remains important to prevent spread, but our findings do not support exclusion of asymptomatic child contacts under 6 years.

## Competing interests

No competing interests are declared by any of the authors.

## Authors’ contributions

LB and JW contributed equally as first authors of the paper including data cleaning, analysis, interpretation and writing of the manuscript. A.P. van Dam conducted the laboratory testing and contributed to the methodology and discussion sections and provided critical review of the paper. GS and A. van den Hoek conceived the research question, provided critical review and guidance throughout and are ultimately responsible for the research. All authors read and approved the final manuscript.

## Pre-publication history

The pre-publication history for this paper can be accessed here:

http://www.biomedcentral.com/1471-2334/12/347/prepub
